# Antistaphylococcal Efficacy of Cefepime, Meropenem, and Piperacillin-Tazobactam in Patients with Polymicrobial Infection with MSSA Bacteremia or Pneumonia

**DOI:** 10.1155/2023/7684613

**Published:** 2023-12-02

**Authors:** Laila M. Najia, Eric Pyles, Arnaldo Lopez-Ruiz, Bibidh Subedi

**Affiliations:** ^1^Department of Pharmacy, AdventHealth Orlando, Orlando, FL 32803, USA; ^2^Department of Critical Care Medicine, AdventHealth Orlando, Orlando, FL 32803, USA

## Abstract

There is a paucity of literature describing de-escalation techniques in patients with polymicrobial infections with one offending organism being methicillin-susceptible *Staphylococcus aureus* (MSSA) being treated with *β*-lactam therapy. The purpose of this study is to determine treatment outcomes for patients with polymicrobial infections with MSSA bacteremia or pneumonia who are treated with cefepime (FEP), meropenem (MEM), or piperacillin-tazobactam (TZP). This trial design represents a retrospective observational three-group comparison study of patients at a community teaching hospital system. Patients reviewed included those who had a MSSA bacteremia or pneumonia in addition to a confirmed polymicrobial infection or presence of a coinfection and received definitive therapy with FEP, MEM, or TZP. The primary outcome is defined as the resolution of fever of ≥100.4°F, hypothermia (≤95°F), leukocytosis (WBC °>° 12,000 cells/mm^3^), and leukopenia with WBC °<° 4,000 cells/mm^3^. Secondary outcomes included duration of definite therapy, in-hospital mortality, hospital and ICU length of stay (LOS), 30-day readmission rates for a presumed infection, and hospital-acquired *Clostridioides difficile* infection (HCDI). From August 1, 2016, to August 30, 2019, 45 patients met eligibility criteria. There were no observed differences in primary endpoint (*p* = 0.65) or secondary endpoints, i.e., in-hospital mortality (*p* = 0.10), hospital LOS (*p* = 0.75), ICU LOS (*p* = 0.53), 30-day readmission rates for presumed infection (*p* = 0.07), or HCDI (*p* = 0.34). There was no difference in treatment success with FEP, MEM, or TZP for polymicrobial infections with one offending organism being MSSA. Due to the lack of evidence in this unique patient population and observed results of our study, randomized studies are warranted to determine appropriate therapy in this complex patient population.

## 1. Introduction

As early as the 1920s, the diagnosis of polymicrobial respiratory infections was described in a literature [1]. In the 1960s, the incidence of *Staphylococcus aureus* pneumonia increased by threefold [1]. Increasing emergence of polymicrobial pneumonia has affected the diagnosing and treatment of community hospital or ventilator-associated pneumonia [2]. Similarly, rates of polymicrobial bloodstream infections, or bacteremia, have more than tripled since the 1970s [3]. Patients with these polymicrobial infections represent a subgroup of hospitalized patients with a high degree of overall complexity and a high risk for morbidity and mortality [2, 3].

Patients with presumed infection typically have empiric antibiotic therapy initiated often consisting of combination of broad-spectrum agents [4]. The transition from empiric to definitive therapy is based on appropriate acquisitions of culture and sensitivity data and safety and efficacy of the antibiotic along with a multitude of other factors [4]. Cefepime (FEP), meropenem (MEM), and piperacillin-tazobactam (TZP) all represent antibiotics within the *β*-lactam class, each providing a broad-spectrum coverage of several Gram-positive and Gram-negative organisms including methicillin-sensitive *Staphylococcus aureus* (MSSA) [5–7]. Advantages of definitive monotherapy compared to combination therapy include less overall antibiotic exposure, decreased risk of antibiotic-related adverse events, and possible decreased risk of antibiotic resistance [8–10].

Antistaphylococcal activity of the *β*-lactam agent is not universal and can vary between agents within the *β*-lactam class. Narrow spectrum *β*-lactams such as cefazolin, oxacillin, or nafcillin are the first-line treatment options for MSSA infections [11]. Currently, there is a lack of literature involving transitioning to monotherapy in polymicrobial infections with MSSA bacteremia or pneumonia. Studies have assessed definitive monotherapy options for patients with diagnosed solely with MSSA bacteremia [12–14], and others have published data on definitive monotherapy in patients with polymicrobial infections [15–17], but no published literature has described clinical outcomes in a cohort of patients who have both MSSA and polymicrobial infections treated with *β*-lactam monotherapy. The aim of this study is to determine if monotherapy with FEP, MEM, or TZP is an effective antibiotic strategy in patients with MSSA bacteremia or pneumonia that is either polymicrobial or accompanied by one or more coinfections.

## 2. Materials and Methods

### 2.1. Study Design

This was a retrospective observational three-group comparison study. This study was conducted at seven community teaching hospital system in the United States. Patients were screened from August 1, 2016, to August 30, 2019, for eligibility. This time period was selected as it was after the publication of the 2016 Infectious Diseases Society of America's hospital-acquired and ventilator-associated pneumonia guidelines [11].

### 2.2. Study Participants

Patients were eligible for inclusion if they were age ≥18 years with positive cultures for MSSA bacteremia or pneumonia, in addition to a confirmed polymicrobial infection or coinfection, who received definitive treatment with FEP, MEM, or TZP for a minimum of four days. Definitive therapy was defined as days of therapy where FEP, MEM, or TZP was the single antibiotic being administered. Antibiotics given polymicrobial infections were defined as ≥2 organisms in one source. Coinfection was defined as ≥2 sources of infection growing ≥1 organism. For inclusion, at the time of empiric therapy, patients must also have one of the following: fever of ≥100.4°F (38°C) or hypothermia ≤95°F (35°C), leukocytosis with WBC >12,000 cells/mm^3^, or leukopenia with WBC <4,000 cells/mm^3^. Patients were excluded if they had methicillin-resistant *Staphylococcus aureus* infections, coinfections limited to uncomplicated skin and soft tissue infection, and primary immunodeficiency defined as patients chronically on immunoglobulin requirements or chronic colony-stimulating factors.

### 2.3. Data Collection

Discern Analytics 2.0™ was used to gather a baseline patients' list for screening. Patient information was cross referenced to study antibiotic use and microbiology results to determine a finalized list. A manual chart review was performed to determine if the patients met eligibility criteria.

### 2.4. Outcomes

Primary objective was treatment success, defined as the resolution of fever or hypothermia and leukocytosis or leukopenia, as adopted by Harris et al. [18]. Secondary objectives included duration of definitive therapy and whether that duration met minimum guideline recommended days of therapy, in-hospital mortality, hospital and ICU length of stay (LOS), 30-day readmission rates for presumed infectious causes, and hospital-acquired *Clostridioides difficile* infection (HCDI). Days of therapy were defined as any day a patient received at least 1 dose of antibiotic. Exploratory secondary outcomes included microbiologic clearance in patients with MSSA bacteremia, defined as no new positive blood cultures once study drug was initiated, and time to septic shock resolution, defined as the time to sustained cessation of vasopressor therapy (for ≥4 hours).

### 2.5. Statistical Analysis

JASP statistical analysis software was used to compute the statistical analyses. Descriptive statistics was used to describe variables in each group. The Shapiro–Wilks test was used to test for normality. The ANOVA test was used to analyze continuous data and Pearson *χ*^2^ for categorical data.

## 3. Results and Discussion

### 3.1. Results

**Figure 1:**
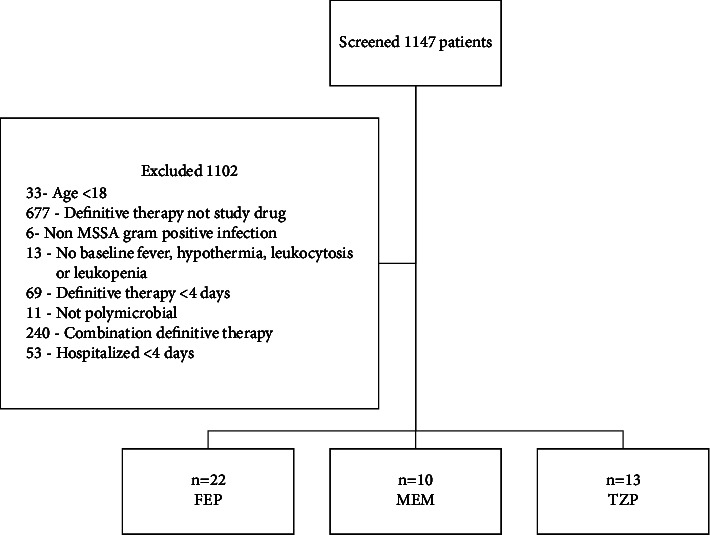
Screening patients. MSSA: methicillin-sensitive *Staphylococcus aureus*; FEP: cefepime; MEM: meropenem; TZP: piperacillin-tazobactam.

From August 1, 2016, to August 30, 2019, 1,147 patients had presented with MSSA bacteremia or pneumonia to our hospital system. Patients were screened for eligibility (Figure 1). Forty-five patients met eligibility criteria. Of them, 22 were in the FEP group (48.9%), 10 were in the MEM group (22.2%), and 13 were in the TZP group (28.9%). Baseline characteristics were well matched between groups (Table 1). Average age was 55 years; 58% were male and mostly white (58%). The most common source of infection was the lung (48.9%) (Table 2). All forty-five patients that were included had MSSA infection. Total number of organisms isolated was 154. The most common organism isolated was MSSA (41.6%) followed by *Pseudomonas spp.* (15.6%) (Table 3). Majority of the patients received FEP, TZP, or MEM as a part of their empiric regimen, and this was continued as definitive therapy as a part of the de-escalation process. The median Charlson comorbidity score was 4.0 (0–8) and was similar between the groups (*p* = 0.74). Thirty-nine patients (87%) were admitted to the ICU with an average SOFA score of 7.5 (3.9–11.1, *p* = 0.73). Thirty-five patients (90%) were on mechanical ventilation and 29 (74%) were on vasopressors during ICU stay. Twelve patients (27%) did not have source control upon admission. Of those patients, 10 (83%) were able to achieve source control and average time to source control was 5.7 days (*p* = 0.84). The primary endpoint of treatment success occurred in 22 patients (48.9%); 7 patients (16%) had missing repeat labs at the end of therapy and could not be assessed (Table 4). There was no difference in treatment success between the groups: FEP 50%, MEM 50%, and TZP 70% (*p* = 0.65). The median duration of definitive therapy did not differ significantly between the groups (*p* = 0.11). Only 8 patients (17.8%) received guideline recommended treatment duration. The median total duration of treatment for both MSSA bacteremia and pneumonia patients was 15 days (5–15), indicating that more patients in the pneumonia group likely received longer duration of therapy than guideline recommendation of 7 days while patients in the MSSA bacteremia group likely received less than guideline recommended duration as it can range from 2 to 6 weeks [11, 19]. Of our 12 patients with bacteremia, 5 patients (41.7%) completed treatment outpatient. We could only assess the duration of treatment for patients who completed therapy inpatient due to the lack of documentation and inability to verify patient's therapy after discharge. In-hospital mortality occurred in 8 patients (17.8%) with no differences between the groups (*p* = 0.10). In patients admitted to the ICU, in-hospital mortality was 20.5%. Patients who expired in the ICU were 15.4%. Our predicted mortality based on the average SOFA score at the time of antibiotic initiation was 27% [20]. Median hospital LOS (*p* = 0.75) and ICU LOS (*p* = 0.53) did not differ significantly between the groups. Nine patients (20%) were readmitted within 30 days. Of those, 4 were due to infectious causes. Only 2 patients had HCDI and both were in the FEP treatment group (*p* = 0.34). Exploratory outcomes, including microbiologic clearance in MSSA bacteremia patients (*p* = 0.30) and time to septic shock resolution in patients who were on vasopressors at the start of antibiotics (*p* = 0.42), did not differ significantly between the groups.

### 3.2. Discussion

We compared clinical outcomes in patients with MSSA bacteremia or pneumonia with either polymicrobial infections or coinfections who received definitive treatment with FEP, MEM, or TZP and found no difference between these three agents. To our knowledge, this is the first report analyzing patients with multiple organisms including MSSA. Three previous studies have researched *β*-lactams treatment options in patients with MSSA bacteremia [2, 13, 14]. A retrospective study compared MSSA bacteremia patients who received definitive therapy with either cefazolin, oxacillin, or nafcillin and found lower mortality in the cefazolin group at 30 days (HR: 0.63, 95% CI: 0.51–0.78, and *p* < 0.001) and 90 days (HR: 0.77, 95% CI: 0.66–0.90, and *p* = 0.001) [12]. Rates of recurrent MSSA infection at days 45–365 did not differ between the groups (HR: 1.13 and 95% CI: 0.94–1.36) [12]. Another retrospective study compared cefazolin to ceftriaxone in patients with MSSA bacteremia and found less rates of clinical failure with cefazolin compared to ceftriaxone (29% vs. 55%, *p* = 0.029) [13]. Lastly, a retrospective study analyzed patients with MSSA bacteremia treated empirically and definitively with oxacillin, nafcillin, cefazolin, TZP, ciprofloxacin, or levofloxacin [14]. In this study, the researchers found that patients in the TZP group had higher rates of 30-day mortality compared to the cohort of patients who received oxacillin, nafcillin, or cefazolin (20.8% vs. 2.1%, HR: 0.10, and 95% CI: 0.01–0.78) [14]. Of note, the authors disclosed that patients who received TZP had higher severity of illness defined by an APACHE III score and were more likely to need ICU level care, indicating potential for selection bias. Overall, these three studies show us average treatment success of cefazolin ranging from 71 to 93% and treatment success of oxacillin and nafcillin ranging from 75 to 95% [12–14]. In our study, we found a treatment success of 50–70% for our patients treated with FEP, MEM, or TZP. While this is lower than the aforementioned studies, previous studies only included single infection secondary to MSSA compared to our study which included more complex patients who had multiple organisms in addition to the MSSA, including more Gram-negative organisms such as *Pseudomonas* spp.

Literature regarding the use of monotherapy in patients with polymicrobial infections is more limited. Smaller studies have assessed cure or mortality rates in polymicrobial infections. One retrospective study compared ICU mortality in patients who received MEM for both empiric and definitive therapy compared to patients who had MEM and de-escalated TZP, FEP, or another *β*-lactam [15]. Patients enrolled had polymicrobial infections, the most common source being the lung (46%), abdominal (31%), or other (23%). Of note, patients were not limited to MSSA as one of the offending organisms. Authors found no difference in mortality between the groups (7% vs. 21%, *p* = 0.12) [15]. This study disclosed that 37 patients (55.2%) had multidrug resistant Gram-negative organisms but did not identify what organisms. In comparison, our study had higher mortality in the combined TZP and FEP group (11.4%) and in the MEM group (40%) and with only 3 patients (1.9%) having multidrug-resistant Gram-negative organism, which may be reflective of sicker patient population, as their patient population had a predicted mortality of 12–25% based off the APACHE II score, and our patient population had a predicted mortality rate of 20–30% based off the SOFA score.

A multicenter, open-label, randomized, parallel-group trial was conducted comparing clinical and microbiological response rates in patients with lower respiratory tract infections, sepsis, or intra-abdominal infections treated with monotherapy with MEM or imipenem/cilastatin [16]. Investigators found no difference between the MEM versus imipenem/cilastatin for overall clinical response rates (difference: 8.9%, 95% CI: −4.2% to 21.9%, and *p* = 0.185) and overall microbiologic rates (67.1% vs. 60.3%, difference: 6.9%, 95% CI: −8.7% to 22.4%, and *p* = 0.389) [16]. About half of the infections included were polymicrobial. We only analyzed microbiologic cure rates in our MSSA bacteremia patients as retrospectively looking at MSSA pneumonia patients was difficult to differentiate between colonization versus infection and could have confounded the results. In our 12 bacteremic patients, 10 (83.3%) had microbiologic clearance. Lastly, a retrospective cohort study was conducted to compare tigecycline to TZP, MEM, or FEP with or without vancomycin or daptomycin in solid-organ transplant patients with polymicrobial intra-abdominal infections [17]. Investigators found that patients treated with TZP, MEM, or FEP were more likely to have clinical cure (72.2% vs. 40.7%, *p* = 0.008) and less adverse events than tigecycline (9.3% vs. 29.6%, *p* = 0.026) [17]. Our study had similar cure rates and we found little adverse events as only 2 patients experienced HCDI, a consequence of antibiotic exposure.

There are several limitations to this study. The retrospective nature of this study can lead to multiple biases and confounding factors that may affect our primary outcome. We attempted to decrease confounding by only assessing outcomes using objective measures. One pitfall was that the definition of source control depended on interpretation from chart review of notes which could have inter-researcher variability. We could only assess patients who completed therapy inpatient for the primary and secondary outcomes and, therefore, may not reflect true long-term outcomes in these patients. We were assessing monotherapy of definitive coverage; however, patients could have received any duration of empiric therapy with dual coverage. The definition of days of therapy did not account for missed doses or whether the patient received all the doses as it was difficult to determine based on the chart review. Finally, the small sample size did not allow for a power calculation; nonetheless, given the complexity of the studied population, this literature provides some insight in characterization of these patients.

## 4. Conclusion

This retrospective study found that patients with MSSA bacteremia or pneumonia along with other organisms had no difference in treatment outcomes when treated with FEP, MEM, or TZP. However, our study was purely observational in nature with several limitations; therefore, further studies are needed to elucidate this specific understudied population to determine the impact of FEP, MEM, or TZP for polymicrobial or coinfections with one offending organism being MSSA.

## Figures and Tables

**Table 1:** Baseline characteristics.

	Overall (*n* = 45)	FEP (*n* = 22)	MEM (*n* = 10)	TZP (*n* = 13)	*p* value
Age, mean (SD)	55.3 (35.9–74.8)	54.9 (37.9–72.0)	58.2 (36.8–79.6)	53.8 (30.8–76.7)	0.86
Gender, male	26 (58)	13 (59)	6 (60)	7 (53.8)	0.94
Race					0.48
White	26 (58)	15 (68.2)	4 (40.0)	7 (53.8)	
Black or African American	9 (20)	5 (22.7)	2 (20)	2 (15.4)	
Others	10 (22)	2 (9.1)	4 (40)	4 (30.8)	
SOFA score, mean (SD)	7.5 (3.9–11.1)	7.9 (4.5–11.4)	7.4 (3.6–11.3)	6.8 (3.0–10.6)	0.73
Charlson comorbidity score, median (IQR)	4.0 (0–8)	3.1 (1.1–5.3)	3.8 (1.1–6.5)	3.5 (0.6–6.5)	0.74
Polymicrobial infection	30 (67)	14 (63.6)	8 (80)	8 (61.5)	0.59
Coinfection	21 (47)	13 (59.1)	3 (30)	6 (46.2)	0.30
ICU admission	39 (87)	21 (95.5)	7 (70.0)	11 (84.6)	0.14
Vasopressors during ICU stay	29 (74)	18 (81.8)	5 (50.0)	6 (46.2)	0.13
Vasopressors at the start of antibiotics	18 (40)	10 (45.5)	4 (40.0)	4 (66.7)	0.33
Mechanical ventilation	35 (90)	18 (81.8)	7 (70.0)	10 (76.9)	0.71
MSSA pneumonia	33 (73)	6 (27.3)	2 (20.0)	4 (30.8)	0.84
No initial source control	12 (27)	6 (50)	3 (25)	3 (25)	0.93
Time to source control, mean (SD)	5.7 (1.4–10)	5.4 (−0.28–11.1)	7 (5–9)	4.5 (−0.5–9.5)	0.84

*n* (%) unless otherwise stated. SD: standard deviation; ICU: intensive care unit, IQR: interquartile range; SOFA: Sequential organ failure assessment; MSSA: methicillin-sensitive *Staphylococcus aureus*; FEP: cefepime; MEM: meropenem; TZP: piperacillin-tazobactam.

**Table 2:** Sources of infection.

Sources (*N* = 92)	*n* (%)
Lung	45 (48.9)
Blood	18 (19.6)
Urine	14 (15.2)
SSTI	9 (9.8)
Abdomen	6 (6.5)

SSTI: skin and soft tissue infection.

**Table 3:** Isolated organisms.

Organisms (*N* = 154)	*n* (%)
MSSA	64 (41.6)
*Pseudomonas* spp.	24 (15.6)
*Enterobacter* spp.	14 (9.1)
*Klebsiella* spp.	12 (7.8)
*Serratia* spp.	6 (3.9)
Coagulase negative staphylococcus	6 (3.9)
*Citrobacter* spp.	4 (2.6)
*Proteus* spp.	4 (2.6)
*Escherichia* spp.	4 (2.6)
Resistant organism^*∗*^	3 (1.9)
Others	13 (8.4)

^*∗*^Resistant organism included *E. coli* ESBL, *P. aeruginosa* MDRO, and *K. pneumoniae* ESBL. MSSA: methicillin-sensitive *Staphylococcus aureus*.

**Table 4:** Endpoints.

	Overall (*n* = 45)	FEP (*n* = 22)	MEM (*n* = 10)	TZP (*n* = 13)	*P* value
Primary endpoint					
Treatment success^*∗*^	22/38 (58)	11/20 (50)	4/8 (50)	7/10 (70)	0.65
Secondary endpoints					
Duration of definitive therapy, median (IQR)	7.0 (1–13)	7.2 (3.7–10.8)	10.2 (5.9–14.5)	8.1 (4.7–11.4)	0.11
Duration of empiric antibiotics, median (IQR)	6.0 (1–11)	9.5 (−2.6–21.7)	7.2 (1.9–12.5)	8.7 (0.43–17.0)	0.83
Received guideline recommended therapy	8 (17.8)	2 (9.1)	2 (20)	4 (30.8)	0.26
In-hospital mortality	8 (17.8)	2 (9.1)	4 (40)	2 (15.4)	0.10
Hospital LOS, median (IQR)	19 (3–35)	26.1 (7.1–45.1)	28.8 (12.1–45.5)	23.2 (6.8–39.5)	0.75
ICU LOS, median (IQR)	13 (−1.5–27.5)	15.2 (3.4–26.9)	20.7 (7.8–33.6)	15.5 (5.7–25.2)	0.53
30-day readmission rates	9 (20)	3 (15)	2 (33.3)	4 (40)	0.47
Readmission due to infectious causes	4 (8.9)	0	2 (100)	2 (50)	0.11
Hospital-acquired CDI	2 (4.4)	2 (10)	0	0	0.34
Exploratory endpoints					
Microbiologic clearance	10/12 (83.3)	5 (83.3)	1 (50)	4 (100)	0.30
Days to septic shock resolution, mean (SD)	3.3 (0.65–6.0)	3.8 (0.6–7.0)	0.9 (−0.1–2.0)	3.8 (4.4–3.1)	0.42

*n* (%) unless otherwise indicated. ^*∗*^treatment success was only calculated in 38 patients as 7 patients had missing labs. IQR: interquartile range; LOS: length of stay; CDI: *Clostridioides difficile* infection; FEP: cefepime; MEM: meropenem; TZP: piperacillin-tazobactam.

## Data Availability

The data that support the findings of this study are available from the corresponding author upon request.
